# How do Different Types of Synesthesia Cluster Together? Implications for Causal Mechanisms

**DOI:** 10.1177/03010066211070761

**Published:** 2022-01-18

**Authors:** Jamie Ward, Julia Simner

**Affiliations:** 1948 University of Sussex, UK; 1948 University of Sussex, UK

**Keywords:** synesthesia/synaesthesia, individual differences, multisensory/cross-modal processing, hearing-motion, mirror-touch, personification

## Abstract

It is unclear whether synesthesia is one condition or many, and this has implications for whether theories should postulate a single cause or multiple independent causes. Study 1 analyses data from a large sample of self-referred synesthetes (*N*  =  2,925), who answered a questionnaire about *N*  =  164 potential types of synesthesia. Clustering and factor analysis methods identified around seven coherent groupings of synesthesia, as well as showing that some common types of synesthesia do not fall into any grouping at all (mirror-touch, hearing-motion, tickertape). There was a residual positive correlation between clusters (they tend to associate rather than compete). Moreover, we observed a “snowball effect” whereby the chances of having a given cluster of synesthesia go up in proportion to the number of other clusters a person has (again suggesting non-independence). Clusters tended to be distinguished by shared concurrent experiences rather than shared triggering stimuli (inducers). We speculate that modulatory feedback pathways from the concurrent to inducers may play a key role in the emergence of synesthesia. Study 2 assessed the external validity of these clusters by showing that they predict performance on other measures known to be linked to synesthesia.

## Introduction

People with synesthesia have unusual, elicited experiences so, for example, numbers may elicit colors and words may elicit taste. Synesthesia emerges in childhood or possibly even earlier (Simner & Bain, 2013) and is regarded as a persistent “condition” or trait that affects a few percent of the population (Simner et al., 2006). Synesthesia likely stems from genetic differences that affect the structural and functional development of the brain (Bargary & Mitchell, 2008). The unusual inner worlds of synesthetes appear normal to them and synesthesia is not linked to general cognitive dysfunction, although it may nevertheless be linked to a distinctive profile of strengths and weaknesses (Rouw & Scholte, 2016). Nevertheless, research into synesthesia is important for several reasons. It can be regarded as a paradigmatic case of neurodiversity, unified by a distinct phenomenology and neurocognitive profile, but without an assumption of pathology (Ward, 2019b). It is also used as a research tool to understand cognitive processes such as language (Root et al., 2021) and perception (Sagiv & Frith, 2013). Nevertheless, there remain key gaps in our understanding that limit research progress. Here we consider one unresolved issue: How many different types of synesthesia are there?

The answer to that question is partly an empirical one, driven by observation, but also a theoretical one relating to how one defines a countable “type” and, indeed, how one defines synesthesia itself (Simner, 2012). The most common approach is to consider types of synesthesia as defined by the pairing of an inducer (the stimulus that elicits it) and concurrent (the synesthetic experience itself), with the convention being to place the inducer first (e.g., “number-color synaesthesia” is induced by numbers and gives rise to unusual colors). Using this kind of approach it has been claimed there are over 60 known types of synesthesia (Day, 2005) and perhaps more than a hundred (Eagleman & Cytowic, 2009). Synesthetes can vary from having one or two of these to being very prolific. However, this does not preclude the possibility of meaningful higher-order groupings. For example, the term “sequence-space synesthesia” refers to a family of different basic types in which sequential concepts (numbers, months, weekdays, etc.) are visualized in as patterns in 2D or 3D space (Eagleman, 2009). Similarly, the term “grapheme-color synesthesia” is sometimes used as an umbrella term for two synesthesias that are elsewhere described separately (number-color, letter-color). But are these groupings meaningful? And are there even greater groupings? For example, should we simply say “visual synaesthesia” as the over-arching family group that incorporates both visuospatial and color concurrents? The answers to these questions are important because they inform our attempts to explain the causes of synesthesia. If different types of synesthesia cluster together then this would be suggestive of a common cause (e.g., common genes, common neural pathways, common environmental bias), whereas separable or partially separable clusters of synesthesia would be suggestive of different causal influences.

Novich et al. (2011) conducted a comprehensive analysis of how different inducer-concurrent pairings cluster together in synesthesia. They analyzed a large set (*N*  =  19,133) of self-referred synesthetes who had indicated the presence or absence of 22 synesthetic pairings (e.g., weekdays-color, vision-smell). Statistical analyses such as clustering of similar correlations and factor analyses suggested that the types of synesthesia fall into five clusters. These were labeled as: colored sequences (e.g., number-color, weekday-color), colored sensations (e.g., pain-color), colored music (e.g., pitch-color), non-visual sequelae synesthesia (e.g., vision-taste), and sequence-space synesthesia. Note that the latter was more of an island than a cluster in that it consisted of a single example (termed “spatialized sequences”) that did not correlate strongly with any other. Subsequent research has provided external validity for this taxonomy: the number of synesthesia clusters that a person reports using this taxonomy (from 1 to 5) correlates with certain measures of cognition and personality (Rouw & Scholte, 2016; van Leeuwen et al., 2019). In effect, there is a link between a more prolific profile of synesthesia and a more extreme behavioral profile.

Novich et al. (2011) speculated that the existence of different clusters of synesthetic types might arise from different genetic causal mechanisms, that is, different genes for different clusters. However, their results are compatible with other interpretations. It is possible that the same genetic influences give rise to, say, both concurrent taste and color experiences but whether one or the other emerges depends on variability in gene expression throughout the brain (favoring taste for one person but color for another) or other influences, such as in the environment, that might drive localized differences (Ward, 2019b). Another argument against the strong view that there are as many causes of synesthesia as there are observable clusters is the fact that different clusters of synesthesia (e.g., sequence-space and colored sequence) present with a relatively similar cognitive profile (Mealor et al., 2016; Ward et al., 2017). Instead, one could perhaps imagine synesthesia existing in three nested levels: a simple presence/absence of synesthesia (i.e., such that all synesthetes have some broad commonalities), an intermediate level of clustered types (such as those identified by Novich et al., 2011), and a basic level of inducer-concurrent pairings (e.g., such that an individual can have days-color but not months-color even if these tend, on average, to cluster together). As a note on terminology we henceforth refer to individual inducer-concurrent pairings as types of synesthesia and refer to *clusters* to denote a group of associated types.

One limitation of Novich et al. (2011) is that they considered only 22 types whereas other estimates suggest there are many more. Notable omissions were mirror-touch synesthesia (Banissy & Ward, 2007), ordinal linguistic personification (Simner & Holenstein, 2007), and hearing-motion synesthesia (Saenz & Koch, 2008). In some of these cases, there have been theoretical debates about whether these are “true” types of synesthesia, that is, whether they share the same defining features as more commonly accepted examples of synesthesia (Fassnidge et al., 2017; Rothen & Meier, 2013). If they were to cluster with well-accepted types and show a similar profile in other respects (e.g., external validity) then this would speak against these reservations (Simner & Holenstein, 2007). Other well-known types of synesthesia, such as lexical-gustatory (Ward & Simner, 2003), were not straightforwardly represented in the 22 inducer-concurrent pairs used by Novich et al. (2011) with sound-taste being the closest option. The present study is an analysis of our own synesthesia questionnaire which was developed primarily as a tool for synesthetes to inform us about the types of synesthesia they possess (from a set of 164 potential types) in order to volunteer for research. Study 1 takes the same statistical approach as Novich et al. (2011) to identify potential clusters of synesthesia. Study 2 establishes the external validity of the clusters identified in Study 1 by showing how these clusters affect other measures known to be related to synesthesia (mental imagery, sensory sensitivity, and projector status of synesthetes with colored letters and/or numbers).

## Study 1: Identification of Synesthesia Clusters

### Method

#### Participants

The participants consist of a large cohort of self-declared synesthetes who have contacted the University of Sussex since 2007 and filled in a survey that documents their types of synesthesia. The inclusion criteria were being aged 18 years or over and reporting one or more potential types of synesthesia, and *N*  =  2,925 participants met these criteria (mean age  =  34.82 years, S.D.  =  12.44, range  =  18–81; 2,421 females, 504 males). These participants form our “Inclusive” dataset because their responses were treated at face value. A subset of *N*  =  2,789 comprised our “Stringent” dataset after various quality control exclusions had been applied, as detailed later (e.g., to remove implausible responses and unlikely synesthetes). Their mean age was 34.76 years (S.D.  =  12.36, range  =  18–81) and they comprised 2,320 women and 469 men. This sex difference is not a meaningful trait of synesthesia, but rather a likely outcome of the greater likelihood of females to self-refer for research (for discussion see, Simner & Carmichael, 2015).

#### Materials and Procedure

The survey was hosted by an online provider (www.onlinesurveys.ac.uk) and accessed via the Synesthesia Research website at the University of Sussex (www.sussex.ac.uk/synaesthesia). The study was approved by the Cross-Schools Research Governance and Ethics Committee. The survey took around 10 min to complete.

Participants were presented with a grid of 19 potential types of inducer in rows (e.g., letters, numbers) and eight types of potential concurrent experiences in columns (e.g., colors, tastes). Participants were instructed: “This grid enables you to tell us about the types of synaesthesia that you may have. The list going DOWN is a list of possible triggers of synaesthesia. The list going ACROSS is a list of possible synaesthetic experiences. So if letters trigger colours for you (i.e., you have letter-to-colour synaesthesia) then you can tick the upper left box; and so on.” The full list of concurrents were: colors, shapes, taste, smell, noise, music, pain, and touch (an “other” option was also included but is not analyzed). The full list of inducers were: letters of the alphabet, English words, foreign words, peoples names, numbers, days of the week, months of the year, voices, pain, touch, body postures, music, noise, smell, taste, color, shape, emotion, and punctuation. Participants could select as many or as few as applied out of a maximum possible of 152 ( = 19  × 8). It is to be noted that not all of these combinations represent known or actual types of synesthesia and some may correspond to normative experiences (e.g., pain-emotion). These were removed from the Stringent dataset using data-driven approaches (because these kinds of responses cluster together).

Additional types of synesthesia, not captured by this grid, were asked about separately. With regards to personification synesthesia, participants were asked “Some people always think of certain things (e.g., numbers) as having a gender (e.g., 5 is male) or a personality (e.g., 6 is bossy). Do you think this applies to you?” Participants then checked items in a grid with four possible inducers (letters, numbers, days, months) together with the concurrents of “personality” or “gender” (i.e., eight possible inducer-concurrent pairs). A further four types of synesthesia were asked about with bespoke questions (including follow-up questions not considered here). These consisted of mirror-touch synesthesia, hearing-motion synesthesia, sequence-space, and ticker-tape synesthesia (see definitions below). The questions were as follows and the accompanying images/videos are in the Supplemental Materials:

- *Sequence-space:* “Some people always experience sequences in a particular spatial arrangement such as in these examples: [line drawing images]. Do you think this applies to you? [Yes/No].”
- *Tickertape:* “For some people, when they hear speech they see the words spelled out in front of them (like reading tickertape). Sometimes it is colored and sometimes not. When you hear someone speaking do you see the words spelled out? [Yes/No].”
- *Mirror-touch:* “Have a look at this clip [movie of man stroked on left cheek]. Did you feel touch on your face in response to seeing this? [Yes/No/I couldn’t play the video].”
- *Hearing-motion:* “Have a look at this clip [dots moving back and forth]. The moving dots are silent, but some people hear something when they see the dots move. Did you hear something? [Yes/No/I couldn’t play the video].”

Thus, in total, the raw data set consists of 164 columns of data (potential types of synesthesia) together with *N* rows of participants. The data itself consists of 1s and 0s denoting the reported presence or absence of this type of synesthesia. For mirror-touch and hearing-motion, there was a small amount of missing data (8.6% and 8.4%, respectively, from the inclusive dataset) for participants who could not observe the video, and these data points were excluded from the analyses.

#### Analyses

All analyses are conducted in R and the script and data files are included in the open science framework (https://osf.io/r24vb/). Two types of analyses were performed. Firstly, we performed correlations between types of synesthesia followed by clustering of similar correlations using the Inclusive dataset. This helped identify rare and implausible clusters of synesthesia that were removed from the Stringent dataset. The initial analysis was then repeated with only the Stringent dataset. Secondly, we performed an exploratory factor analysis on the Stringent dataset.

In a first analysis, all potential types (*N*  =  164) were correlated together with Pearson's *r*. Hierarchical clustering was then performed based on Euclidean distances between correlations using the (default) complete-linkage method. For example, if letter-color, number-color, and days-color synesthesia all correlated strongly with each other (but correlated weakly with other types of synesthesia) then these would be grouped together and, conceptually, this could be regarded as a synesthesia cluster. The resulting data was visualized as a heatmap (a rearranged correlation matrix, grouping strong patterns of correlation together) and dendrogram (showing the hierarchical relationship of clusters). The number of clusters in this approach is not fixed or pre-determined. However, visualizing a scree plot (plotting height of the dendogram against a number of clusters) provides constraints on a plausible solution. Although it is common practice to use Pearson's correlation as a distance metric, transformations of this value based on logarithms (e.g., Fisher's *r*-to-*z*) or square roots (Solo, 2019) are considered as more appropriate by some researchers and would, if anything, have the positive effect of accentuating larger correlations and diminishing the distance between smaller ones.

This analysis also determined how we came to select our Stringent Dataset (i.e., a dataset reduced from our initial total sample by quality control). We began with an initial analysis of the Inclusive Dataset (i.e., all potential synesthesia). This identified several small clusters that contained scientifically uninteresting pairings (e.g., voices-noise) and may reflect a misunderstanding of the requirements (which itself may be indicative of not having synesthesia). These comprised the following responses: tastes-taste, smells-smell, noises-noise, music-music, color-color, shapes-shape (cluster A); smells-taste, tastes-smell, voices-noise, voices-music, noises-music, music-noise (cluster B); body postures-shape, punctuation-shape, letter-shape, number-shape, peoples name-shape, English words-shape, and foreign words-shape (cluster C). Participants who had given a high proportion of these responses were excluded entirely (*N*  =  127). Specifically, the upper 5% of the sample were removed, corresponding to seven or more endorsements of these response options. Furthermore, these inducer-concurrent pairings were excluded together with four others, which had not clustered but were produced in a similar vein (pain-pain, touch-touch, pain-touch, touch-pain). Finally, we excluded any very rare types of synesthesia (<1% prevalence amongst candidate synesthetes; e.g., punctuation-music, months-pain, tastes-noise). After excluding these very rare or implausible types of synesthesia, some participants (*N*  =  9) had no types of synesthesia at all and were excluded. Hence, the Stringent Dataset considers 2,789 participants (down from 2,925) and 112 potential types of synesthesia (down from 164). This was then reanalyzed in terms of correlations and clustering (as described for the Stringent Dataset), and exploratory factor analysis.

A Maximum Likelihood Factor Analysis, with Varimax rotation, was conducted on the stringent dataset. Kaiser-Meyer-Olkin (KMO) Measure of Sampling Adequacy and Bartlett's Test of Sphericity were used to check if this method is appropriate, where the KMO should be above 0.6 and Bartlett's test should be significant, *p* < .05 (Williams et al., 2010). The number of participants to variables exceeded the common guidelines of 10:1. The number of factors to extract was informed by considering the eigenvalues (variance explained ranked from maximum to minimum) and performing a parallel analysis, PA, to identify the number of factors (Hayton et al., 2004). To mitigate over-extraction in PA, an alpha will be set at 0.01 (99% percentile).

### Results

#### Preliminary Analysis: Most Prevalent Types

**Figure 1.**
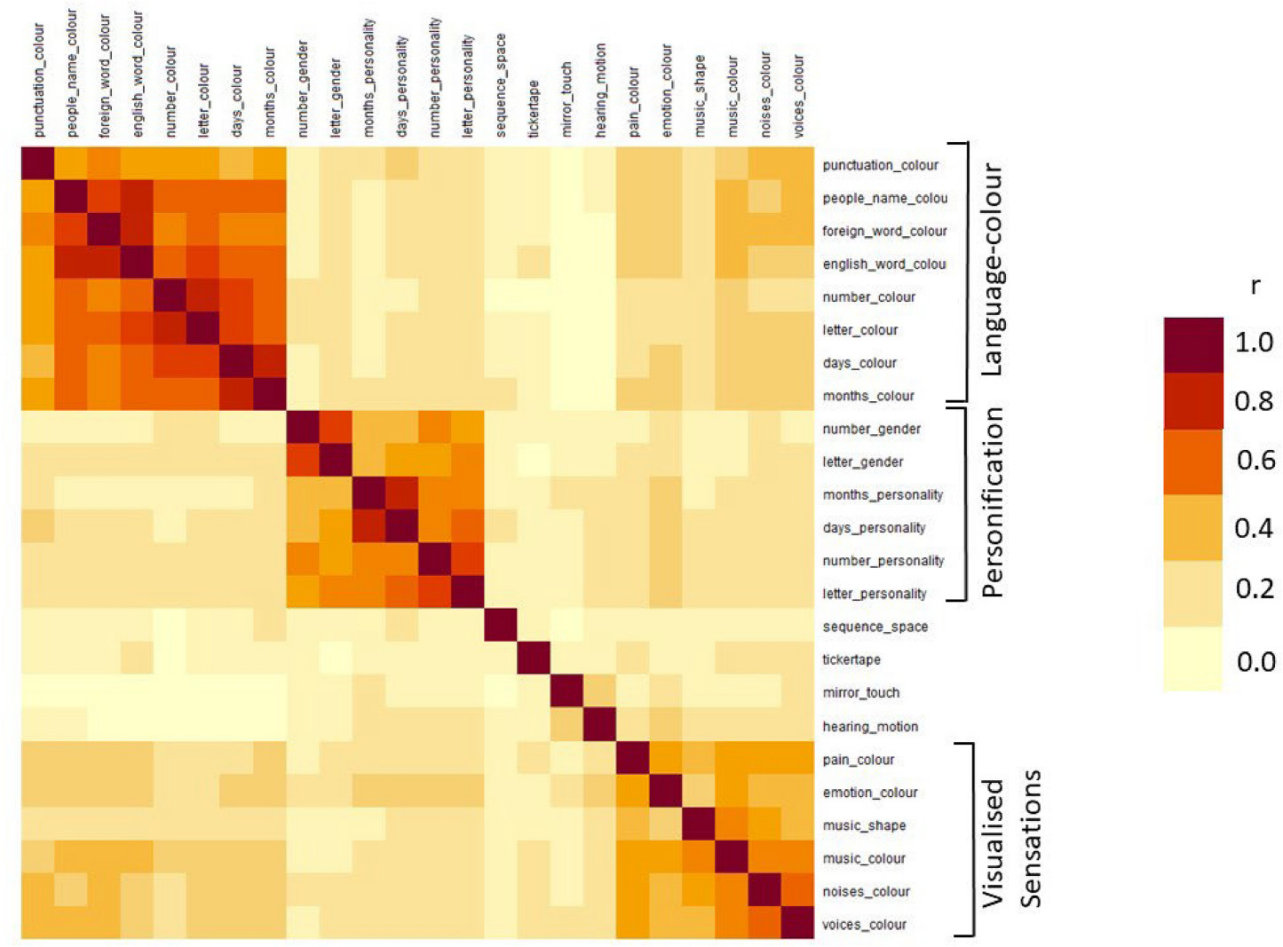
The correlation matrix amongst the most common types of synesthesia (prevalence amongst synesthetes of at least 20%) reveals three clusters (Language-color, Personification, Visualized Sensations) and four “islands” that do not correlate strongly with any other common type (sequence-space, tickertape, mirror-touch, hearing-motion).

There were 24 types of synesthesia with a prevalence of >20% (common to both Inclusive and Stringent Datasets). Their correlations are shown in Figure 1. Amongst these common types, there were three apparent clusters: one consists of what we might call Language-Colour synesthesia (e.g., letter-color, English word-color, *N* = 8 types), another encompassed what we might call Visualized Sensation (e.g., pain-color, music-shape, *N*  =  6 types), and the third was Personifications (e.g., number-gender, days-personality, *N  =  *6 types). Four of the prevalent types of synesthesia did not cluster together with any other in this set: tickertape, hearing-motion, mirror-touch, and sequence-space synesthesia. A more formal analysis of the clusters, including all possible types of synesthesia, is given below.

#### Correlations and Hierarchical Clustering of Types of Synesthesia

**Figure 2.**
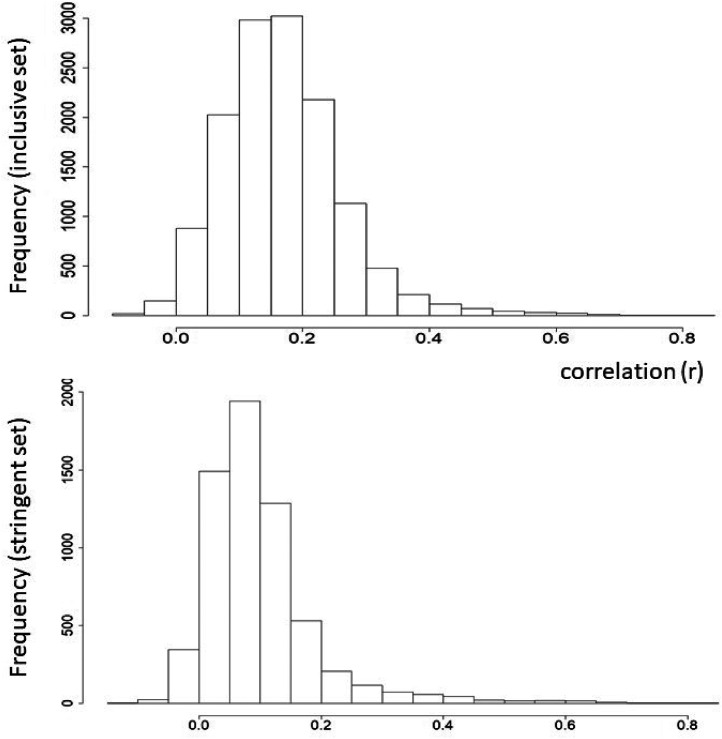
Histograms showing the distribution of correlation coefficients (*r*) between all types of synesthesia from the Inclusive dataset (top) and Stringent dataset (bottom). The mean and SD of the Inclusive dataset is 0.167 (0.095). The mean and SD of the Stringent dataset is 0.100 (0.099)

There are (164  × 163)/2 unique correlations (*N*  =  13,366) for the inclusive dataset and (112  × 111)/2  =  6,216 for the stringent dataset. The distribution of correlations is shown in Figure 2 and demonstrates an overall set of positive correlations amongst types of synesthesia (pairs of inducers-concurrents), with the majority being only weakly correlated (i.e., *r *< .3 being indicative of a small effect size). The absence of any notable negative correlations implies that types are not competing against each other.

**Figure 3.**
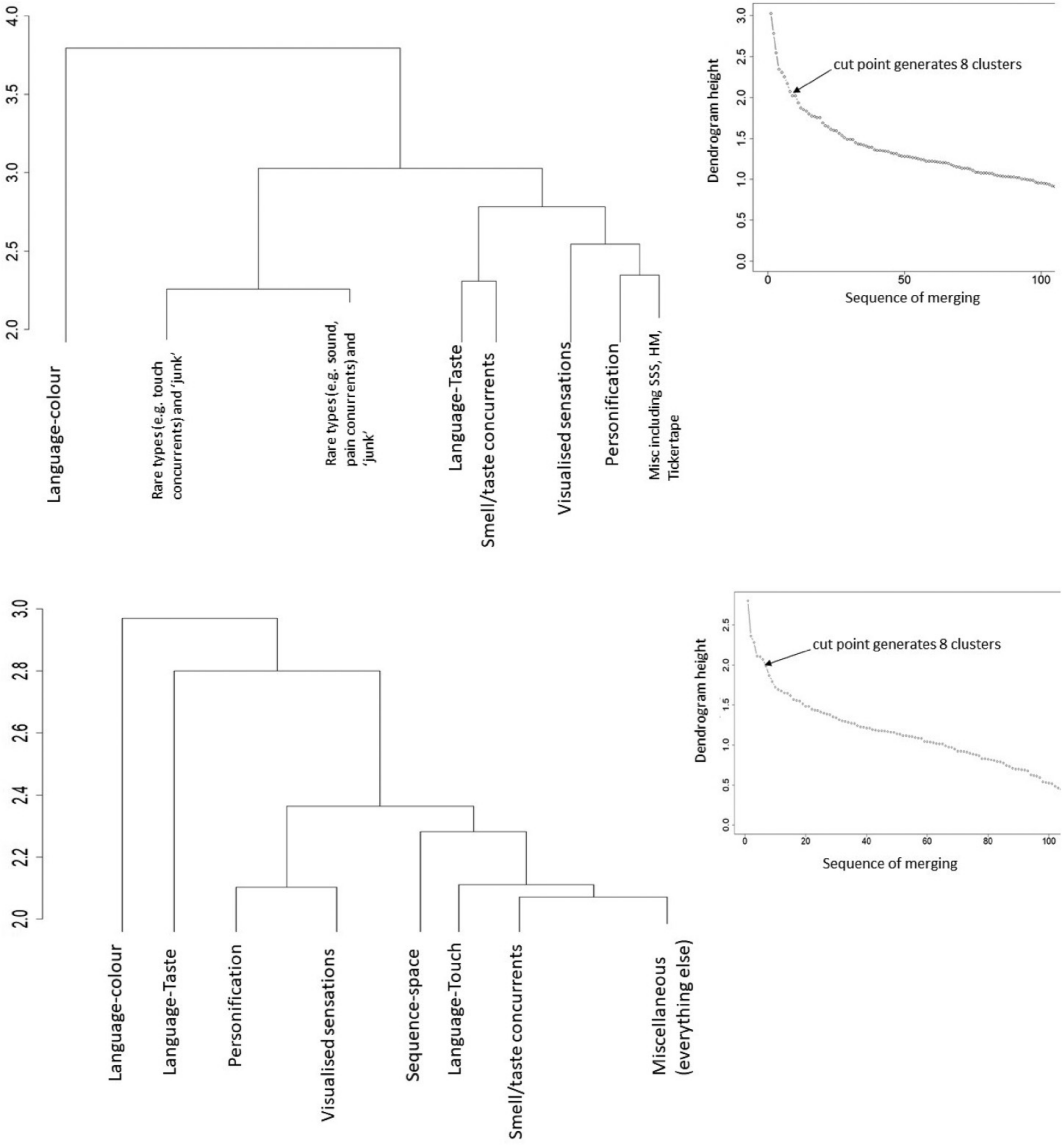
Dendrograms and scree plots for the inclusive dataset (top) and stringent dataset (bottom). The tree is cut at eight clusters (where the eighth cluster represents all types that are leftover and is labeled here as miscellaneous). The full dendrgrams showing all *N*  =  164 and *N*  =  112 types are shown in supplementary results from which the results of different cuts can be inferred.

The pattern of correlations was visualized as dendrograms based on hierarchical distance-based clustering. Figure 3 shows the results of the hierarchical clustering when extracting the first eight clusters, for both the Inclusive and Stringent Datasets (see below for why eight were chosen; and see supplementary material for their full dendrograms of *N*  =  164 and *N*  =  112 pairings, respectively). The left-right ordering on the dendrogram corresponds to the tightness of the clusters, such that the rightmost (i.e., eighth) cluster inevitably contains pairings that display little or no clustering at all (i.e., islands). In both datasets, three of the most prevalent types of synesthesia—mirror-touch, hearing-motion, and tickertape—all reside in that category. Clusters that join together towards the bottom of the dendrogram (e.g., Visualized Sensations and Personification) have correlations between each other that are numerically closer, and clusters that join together at the higher levels (e.g., Language-Color and Language-Taste) have correlations between each other that are numerically distant. Considering the Inclusive dataset, five of the eight clusters were conceptually coherent insofar as they contained either a set of inducers of the same kind (e.g., linguistic stimuli) or a set of concurrents of the same kind (e.g., color). This applied to seven out of the eight clusters in the stringent set. In the Inclusive set, there are a relatively small group of participants who give dubious answers (e.g., pain-emotion, taste-smell) but—because these answers co-occur together—they give high correlations and they occupy a fairly prominent position in the dendrogram (second and third branches from the left). These were either removed by quality control checks when creating the Stringent Dataset or otherwise migrated themselves to the miscellaneous cluster (by virtue of having only weak correlations with the remaining types).

**Figure 4.**
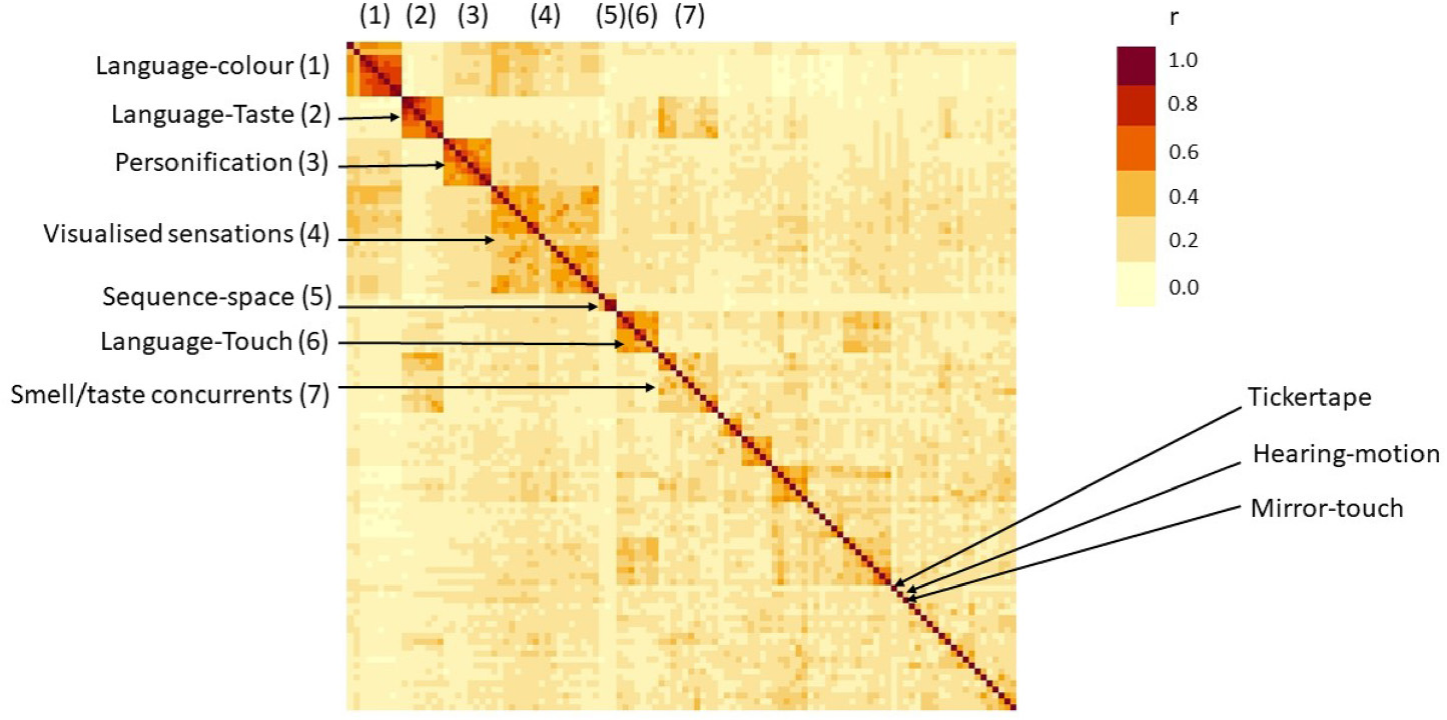
Correlation matrix for the stringent dataset (*N*  =  112 types of synesthesia), ordered according to hierarchical distance-based clustering. The first seven clusters are shown, together with the three most prevalent types within the residual eighth cluster.

The *number* of clusters to extract was informed by a scree plot: plotting height of the dendogram against a number of clusters. Here, the height of the dendrogram represents the distance between the clusters; a higher distance indicates less similarity. In effect, one is searching for a transition point in which adding more and more clusters explains less and less of the data. It would be possible to extract more than eight clusters, and this could indeed be justified from the scree plot for the stringent dataset. However, even doubling the number of clusters would leave the seven leftmost clusters intact, and simply dissect the miscellaneous category (made up predominantly of rare types). If one were to extract further clusters from the Stringent dataset then the eighth clusters would consist of Language-sound (e.g., English word-noise, Foreign word-music; *N*  =  9 types with two sub-clusters of *N*  =  4 and *N*  =  5), followed by a ninth cluster of emotion inducers (e.g., emotion-smell, emotion-pain emotion-music; *N*  =  6 types) and a tenth of pain/tactile concurrents (e.g., smells-pain, color-touch, noises-touch; *N*  =  14 types) noting that the latter does not include mirror-touch synesthesia. Figure 4 shows the correlation matrix of the Stringent dataset with the first seven clusters marked (and remaining candidate clusters visible). It is to be noted that the first five clusters extracted are well documented in the literature and, with the exception of the Language-taste cluster, have a high prevalence. Clusters 6–10 are less well-documented and not all are strong candidates for being genuine kinds of synesthesia (e.g., Language-sound).

The specific types of synesthesia that belong within the first eight clusters are listed in Table 1, together with the prevalence of types and clusters. The data here is taken from the Stringent dataset because we have more confidence in the data quality and it excludes more implausible types.

**Table 1.** The first eight clusters from the Stringent dataset and their associated types. * not from the main grid but from the separate question.

Cluster	Cluster prevalence	Types (type prevalence)
(1) Language-Color	0.684	Number-color (0.547), Letter-color (0.541), Days-color (0.521), Months-color (0.503), People's names-color (0.435), English words-color (0.416), Foreign words-color (0.335), Punctuation-color (0.197), Shapes-color (0.162)
(2) Language-Taste	0.087	English words-Taste (0.065), People's names-Taste (0.048), Foreign words-Taste (0.040), Months-Taste (0.026), Days-Taste (0.022), Letter-Taste (0.023), Number-Taste (0.022)
(3) Personification	0.440	Number-personality (0.302), Number-gender (0.244), Letter-personality (0.229), Letter-gender (0.202), Months-personality (0.197), Days-personality (0.190), Months-gender (0.148), Days-gender (0.128)
(4) Visualized sensations	0.576	Music-color (0.372), Emotion-color (0.298), Music-shape (0.234), Noises-color (0.214), Pain-color (0.193), Voices-color (0.187), Smells-color (0.157), Noises-shape (0.148), Tastes-color (0.128), Voices-shape (0.107), Touch-color (0.104), Pain-shape (0.096), Emotion-shape (0.076), Touch-shape (0.060), Smells-shape (0.053), Tastes-shape (0.054), Body posture-color (0.041)
(5) Sequence-space synesthesia	0.618	Sequence-space synesthesia (0.605*), Days-shape (0.160), Months-shape (0.160)
(6) Language-touch	0.064	English words-touch (0.032), Peoples names-touch (0.031), Foreign words-touch (0.023), Number-touch (0.019), Letter-touch (0.022), Days-touch (0.015), Months-touch (0.015)
(7) Smell/taste concurrents	0.105	Music-taste (0.039), Voices-taste (0.028), Music-smell (0.027), Noises-taste (0.024), English words-smell (0.021), Months-smell (0.020), Peoples names-smell (0.018), Noises-smell (0.015), Foreign words-smell (0.014), Voices-smell (0.012)
(8) Miscellaneous/unclustered	N/A	Hearing-motion (0.366*), Tickertape (0.290*), Mirror-touch (0.288*), Music-touch (0.089), Noises-pain (0.075), Emotion-pain (0.068), Emotion-touch (0.064), Noises-touch (0.064), Voices-touch (0.062), Color-shape (0.057), Emotion-music (0.052), Color-taste (0.051), Color-music (0.045), Pain-noise (0.037), Emotion-taste (0.034), Emotion-smell (0.034), Emotion-noise (0.033), Music-pain (0.033), Color-touch (0.029), Shapes-touch (0.028), English word-noise (0.025), Color-smell (0.025), Touch-noise (0.024), Color-noise (0.024), Voices-pain (0.023), Body postures-touch (0.023), Smells-touch (0.023), Pain-taste (0.023), Tastes-touch (0.022), Punctuation-noise (0.021). There were a further 22 unclustered types with a prevalence < 2% not reported here (see dendrogram in Supplemental Material for details).

#### Factor Analysis

The KMO of .87 suggests that a factor analysis was feasible and Bartlett's Test of Sphericity was significant (Williams et al., 2010). A Maximum Likelihood Factor Analysis, with Varimax rotation, revealed up to 21 factors to extract (based on a parallel analysis) explaining 43.9% of cumulative variance. These are shown in Supplemental Material. The first seven factors (i.e., accounting for the most variance) were largely the same as those derived from the cluster analysis. Factors 1 through to 7 were labeled as Visualized Sensations, Language-Color, Language-Taste, Personification, Language-Touch, Tactile concurrents, and Smell/Taste concurrents (explaining a cumulative variance of 24.7%). A few other factors emerged that were conceptually coherent (e.g., Factor 8 consists of emotion-based inducers, and Factor 11 is Sequence-Space) but most subsequent factors duplicated earlier ones (e.g., Factor 17 consisted of days-personality and months-personality, which were a subcomponent of Factor 4 comprising gender and personality personification more broadly). It is noteworthy that neither tickertape, mirror-touch, nor hearing-motion were grouped into any of the 21 factors (based on factor scores > .3).

In short, the factor analysis produces essentially the same groupings as noted earlier but does not yield a definitive answer as to how many distinct clusters of synesthesia there are. In both cases, the most robust clusters/factors are Language-color, Visualized sensations, Personification, Language-taste, and Language-touch (all except the latter are well documented in the literature). Sequence-space has low correlations with other types.

It is noteworthy that the overall variance explained is low (24.7% across the first seven factors). This reflects sizable residual heterogeneity within the clusters themselves (i.e., at the level of inducer-concurrent pairings). For example, the cluster of Visualized Sensations contains a variety of disparate inducers (pain, music, smell) that rarely all co-occur together but nevertheless have some meaningful degree of association (e.g., a person with one of these inducers is likely to have a second or third inducer from within that cluster). There may also be variability in reporting that does not reflect genuine phenomenological differences. For instance, some people with grapheme-color may not report word-color (because words are colored by graphemes) whereas others report it as a separate kind.

#### Correlations Between Synesthetic Clusters

This analysis describes the relationship between the candidate *clusters* of synesthesia in contrast to previous analysis which focused on correlations between *types* (i.e., individual inducer-concurrent pairings). This involves recoding the data such that each cluster for each participant is coded as 1 or 0 depending on whether there is evidence for that cluster being present or absent (irrespective of how many types within the cluster are observed to be present). We consider the first seven clusters extracted from the analysis of the Stringent Dataset, and also consider the three most common types from within the remaining miscellaneous pool (hearing-motion, mirror-touch, tickertape). The correlations based on the Stringent Dataset are shown in Table 2. Of the 45 possible correlations (i.e., 10  × 9/2) all of them are positive-going and all but one are weak (i.e., *r* < .3). The exception was the correlation between language-taste and smell-taste concurrents (*r*  =  .452). Note that for a sample of this size, all correlations of *r *> .04 are significant at *p* < .05. A sign test shows that the chances of having so many positive correlations (assuming a null distribution centered on zero) is *p* < .001. As such, there is a general positive manifold across clusters of synesthesia and this extends both to the seven true clusters and the three islands.

**Table 2.** Correlations between the first seven extracted clusters from the Stringent dataset (1–7) together with three other common types of synesthesia that do not cluster (8–10). The color key is the same as in Figure 1.

	(1)	(2)	(3)	(4)	(5)	(6)	(7)	(8)	(9)	(10)
Personification (1)		0.133	0.185	0.210	0.055	0.121	0.111	0.181	0.074	0.118
Sequence-space (2)			0.092	0.085	0.012	0.037	0.047	0.117	0.121	0.027
Language-color (3)				0.285	0.029	0.047	0.040	0.030	0.075	0.007
Visualized sensations (4)					0.079	0.186	0.119	0.229	0.115	0.112
Language-taste (5)						0.450	0.194	0.080	0.042	0.107
Smell-taste concurrents (6)							0.228	0.143	0.085	0.183
Language-touch (7)								0.141	0.064	0.133
Hearing-motion (8)									0.116	0.216
Tickertape (9)										0.091
Mirror-touch (10)										

#### Individual Differences in the Number of Clusters Reported

**Figure 5.**
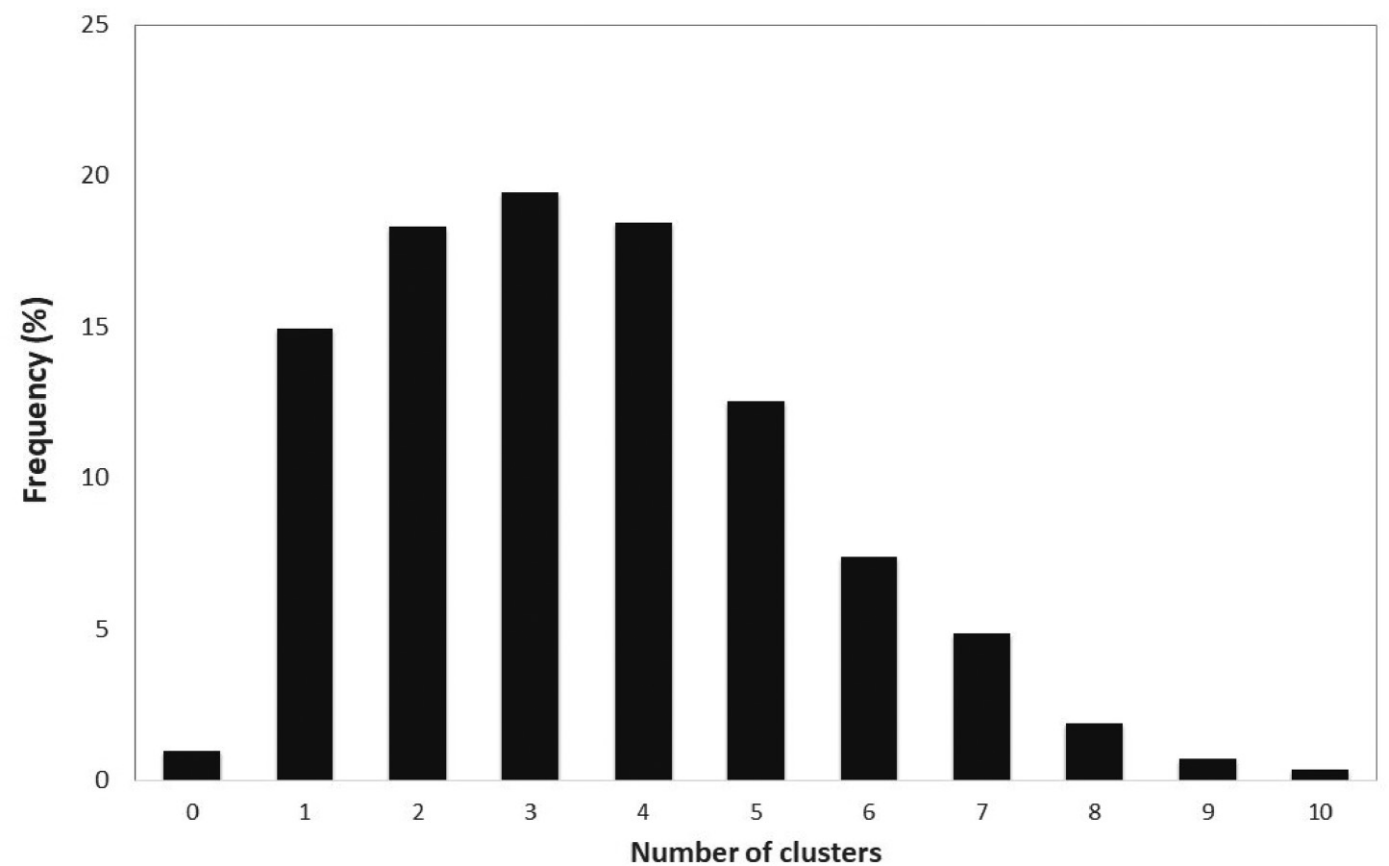
The frequency (%) of the number of clusters of synesthesia reported considering the following list: Language-color, Language-taste, Personification, Visualized sensations, Sequence-space, Language-touch, Smell/Taste concurrents, Hearing-motion, Tickertape, Mirror-touch.

Across these ten clusters/types, the synesthetes from the Stringent Dataset have on average 3.462 (S.D. = 1.888) clusters present. The distribution is shown in Figure 5. A value of zero is possible if a participant declares having types of synesthesia that don’t fall into the main clusters (e.g., Touch-noise is unclustered) and if they don’t additionally report any hearing-motion, mirror-touch, or tickertape. This is rare (*N*  =  25 “synaesthetes”).

**Figure 6.**
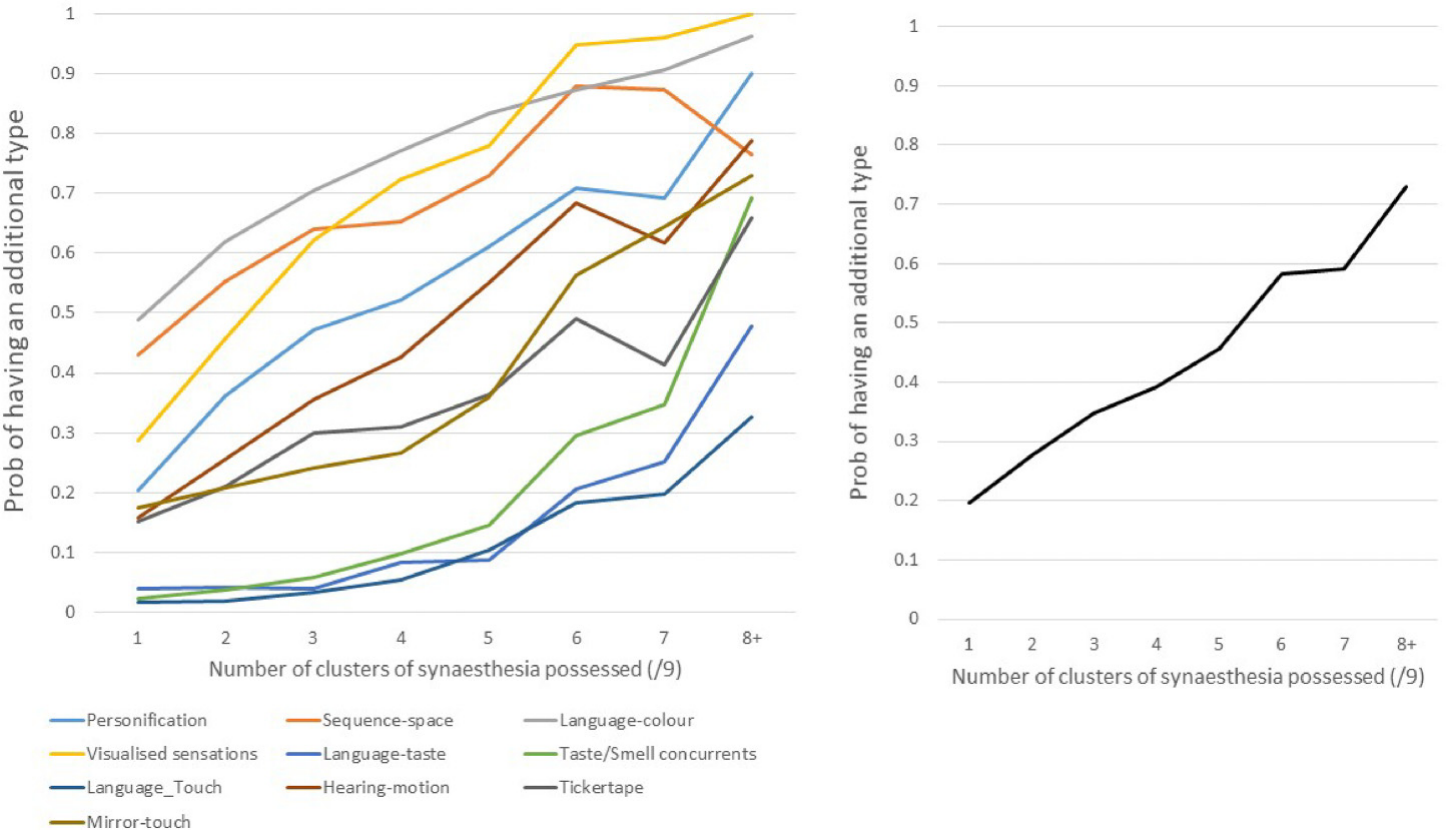
Left: The results of a jackknife procedure in which one cluster of synesthesia is excluded in turn and the number of remaining clusters that a person possesses is counted. The probability of having the excluded cluster (*y*-axis) depends on how many other clusters they have (*x*-axis). The correlations (*r*) between *x*- and *y*-values are: Personification  =  0.294, Sequence-space  =  0.170, Language-color  =  0.202, Visualized sensation  =  0.351, Language-taste  =  0.201, Taste/smell concurrents  =  0.308, Language-touch  =  0.238, hearing-motion  =  0.307, tickertape  =  0.189, mirror-touch  =  0.223. Right: the average of the ten lines plotted in the top figure. This shows the overall “snowball effect” whereby more begets more.

If each cluster had an independent cause then having multiple clusters of synesthesia would be down to chance. The alternative scenario is that having multiple clusters of synesthesia is not a chance event but is itself indicative of a single common cause which results in the proliferation of synesthesia (a snowball effect). Figure 6 shows that a good predictor of whether a synesthete will have any given cluster of synesthesia (*x*) is how many other clusters of synesthesia they have (excluding *x*). This considers the 10 clusters leaving each cluster out in turn and counting the number of remaining clusters that a person has (between 0 and 9), that is, using a jackknife procedure (Nisbet et al., 2018). The fact that none of the lines in Figure 6 are flat (*r* values range from .170 [sequence-space] to .350 [visualized sensations], all *p* < .05) speaks to the claim that these clusters are not fully independent. Note that there is an apparent order to their emergence. For example, visualized sensations become particularly prevalent after 3–4 other clusters are reported but taste–smell concurrents, language-taste, and language-touch tend to only become prevalent at 7  +  .

### Discussion

Through clustering and factor analysis we have been able to identify patterns in the way types of synesthesia group together. These clusters, once extracted, are weakly correlated to each other (tending to correlate at around *r*  =  .1). We argue that these clusters are unlikely to be independent phenomena with wholly independent causes, and demonstrate that any given cluster becomes more prevalent as a function of the number of other clusters a person has—which we term a snowball effect. In the second study, we establish external validity by linking the results of the analysis in Study 1 with measures shown previously to be related to the number of clusters of synesthesia a person has.

## Study 2: External Validity of Candidate Synesthesia Clusters

Previous research has shown that the number of kinds of synesthesia an individual has is related to a number of cognitive traits such as personality (Rouw & Scholte, 2016), sensory sensitivity (Ward et al., 2018a), mental imagery (Spiller et al., 2015), and also related to characteristics of synesthetic phenomenology such as the extent to which the concurrent feels to be located outside (vs. inside) the body (Ward, 2019b). Previously, we have shown a “dose effect,” in which these traits correlate with the amount of synesthesia (e.g., a higher “dose” of synesthesia means greater imagery; Spiller et al., 2015). However, different studies used different methods for counting the dose (e.g., counting types of synesthesia vs. clusters). Here we turn this approach on its head, to test whether the degree of correlation between synesthesia and other traits provide some external validity for a given counting approach. Does extracting 20 clusters of synesthesia help to explain these traits more than extracting only 5 or 10 clusters? Our assumption is that after the “correct” number of clusters has been reached then adding further information will not improve the result and may even hinder it (essentially adding noise to the data). Here we make use of three datasets generated by research from our lab (a mixture of published and unpublished data) and reanalyze them making use of the novel clustering analysis conducted in Study 1.

### Method

#### Participants

Our participants were a large cohort of synesthetes (see below for Ns) who had: (a) taken the synesthesia questionnaire described in Study 1; (b) passed an objective test to validate their synesthesia; and (c) taken other questionnaire measures which have previously been reported to show dose effects. For validation, we selected the “gold standard” measure, in the form of a consistency test (Rich et al., 2005). In a typical consistency test, participants are shown a set of inducers (e.g., letters) and are required to indicate their concurrents (e.g., select the color for each inducer from a color palette). The entire set of inducers is then shown again in a surprise retest, and the dependent measure is the consistency of responding (e.g., was the letter A assigned the same color in both test and retest? Was the letter B? etc.?). Synesthetes must score significantly higher in their consistency compared to controls, on the assumption that synesthetes have fixed colors, while controls answer essentially randomly. Our synesthetes were verified for either grapheme-color synesthesia (Rothen et al., 2013) or sequence space synesthesia (Ward et al., 2018b). It is important to note, however, that these same participants often report other types and the number of clustered types is the variable of interest here. As part of other ongoing research they had also completed one or more of the following tests:

1. Glasgow Sensory Questionnaire (GSQ; Robertson & Simmons, 2013). *N*  =  198 of whom *N* = 181 were published in Ward et al. (2018a). The mean age was 35.328 years (S.D.  =  9.744) and there were 171 females and 27 males.
2. Plymouth Sensory Imagery Questionnaire (PSI-Q; Andrade et al., 2014; short version). *N*  =  203 of whom *N*  =  101 were published in Ward and Filiz (2020). The mean age was 35.291 years (S.D.  =  11.794) and there were 183 females and 20 males.
3. Colored letters and numbers questionnaire (CLaN; Rothen et al., 2013). *N*  =  518 with a mean age of 34.116 (S.D.  =  11.622) and there were 452 females and 66 males.

#### Materials

The *Glasgow Sensory Questionnaire* (GSQ) is a measure of sensory sensitivity, which includes both hyper- and hypo-sensitivity (Robertson & Simmons, 2013). Hyper-sensitivity is over-responsiveness to sensory stimuli (e.g., finding bright lights too glaring), while hypo-sensitivity is under-responsiveness (i.e., sensory “dampening,” often leading to sensory-seeking behaviors such as “stimming”). The GSQ contains 42 items across seven sense domains (auditory, gustatory, olfactory, proprioceptive, tactile, vestibular, and visual) with six items per sense. Within each sense, half of the items (*n*  =  3) measure hyper-sensitivity and half measure hypo-sensitivity. Examples items include “Do bright lights ever hurt your eyes/cause a headache?” (visual/hyper-sensitivity) and “Do you really like listening to certain sounds (e.g., the sound of paper rustling)” (auditory/hypo-sensitivity). Items are rated on a scale of 0 (“Never”), 1 (“Rarely”), 2 (“Sometimes”), 3 (“Often”), and 4 (“Always”). An overall sensitivity score is summed across all items (ranging 0–168), and there are sub-scores for each of the seven senses (e.g., auditory; ranging 0–24). It also produces two scores collapsed over senses for hypo- and hyper-sensitivity respectively (ranging from 0 to 84 each).

The *Plymouth Sensory Imagery Questionnaire* (Psi-Q) is a measure of mental imagery across seven domains in its long-form; visual imagery, auditory imagery, etc. (Andrade et al., 2014). Here we used a shortened version of this comprising only five of the domains (vision, audition, touch, taste, smell) and the top two loading questions on each domain (Ward & Filiz, 2020). Participants were asked to form a mental image involving different senses: auditory (e.g., imagine the sound of a car horn), visual (e.g., imagine the appearance of a bonfire), gustatory (e.g., imagine the taste of black pepper), olfactory (e.g., imagine the smell of burning wood), tactile (e.g., imagine touching fur). Participants rated each item on a scale from 0 (“No image at all”) to 10 (“Image as clear and vivid as real life”). This questionnaire provided imagery scores for each sense domain and an overall average, with possible scores 0–10.

The *Colored Letters and Numbers questionnaire* (CLaN) measures various aspects of the phenomenological experience of grapheme-color synesthesia (Rothen et al., 2013). Here we considered 13 items derived from three subscales (omitting the subscale about longitudinal change): localization (e.g., *I can point to the location of the synesthetic colors*), automaticity/attention (e.g., *The synesthetic colors appear automatically without any effort on my part*), and deliberate use (e.g., *I deliberately try to use my synesthetic colors in my everyday life*), and longitudinal changes (e.g., *My synesthetic colors did not change their intensity over the years*). These factors were externally validated with tests that are widely used in the field of synesthesia research (Rothen et al., 2013). The questionnaire shows good construct validity and test-retest reliability (Rothen et al., 2013), and more extreme scores on the CLaN are also related to the number of other kinds of synesthesia a person reports (Ward, 2019a, 2019b). Responses are given on a 5-point Likert scale from 1 to 5 (i.e., strongly disagree, moderately disagree, neither agree nor disagree, moderately agree, strongly agree) with reverse coding applied and the sum across all items calculated for each synesthete.

### Results and Discussion

**Figure 7.**
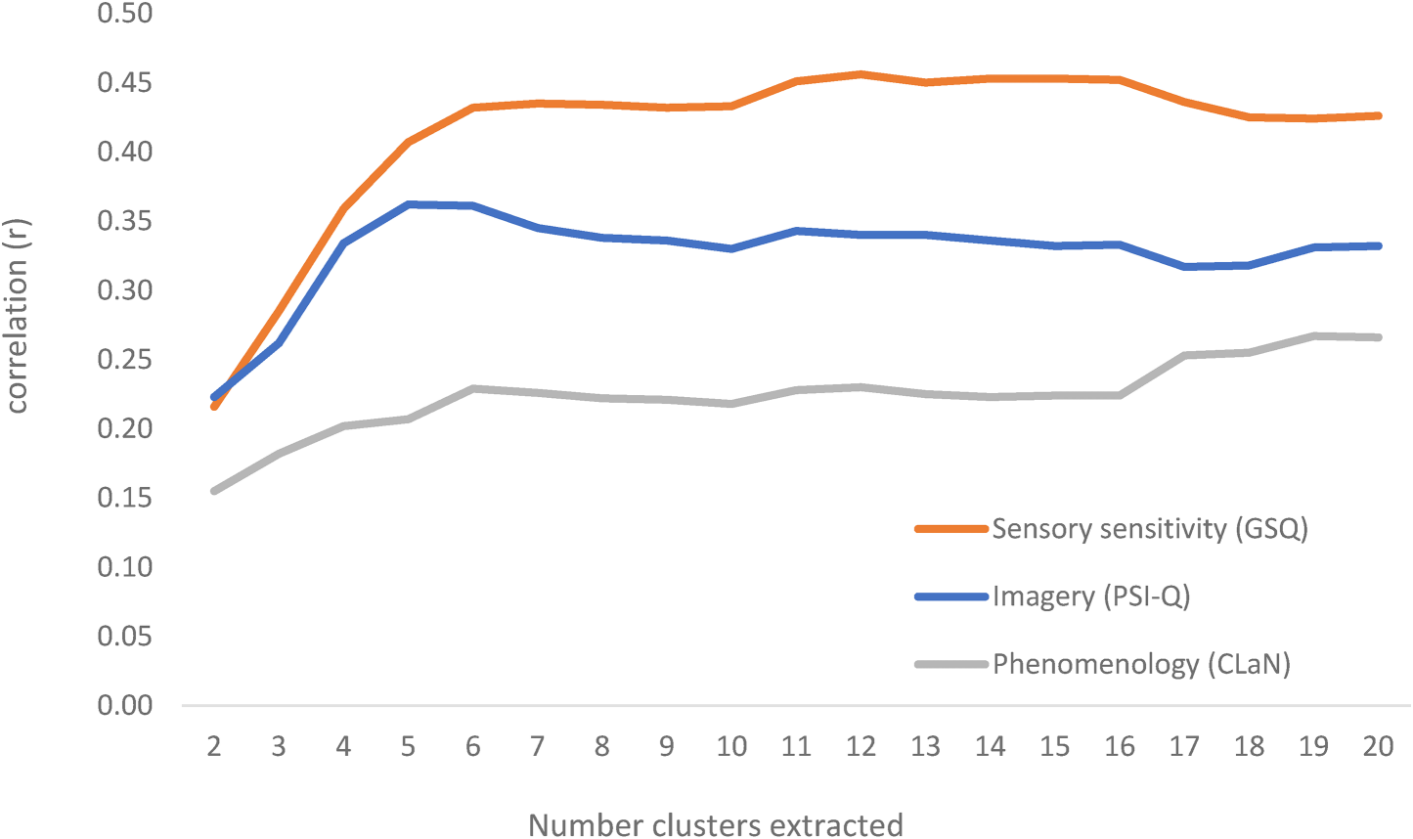
The *x*-axis shows the number of clusters extracted (not the number of types of synesthesia a person has). The correlation (*y*-axis) is calculated between the number of clusters of synesthesia a person has (for a given number of types extracted) against the appropriate questionnaire measure.

Recall from Study 1 that the number of clusters to extract is not strictly determined by the data and has an element of human judgment. Here, the number of clusters to extract is systematically varied and the external validity (the magnitude of the observed “dose effect”) guides the judgment. The clusters derived from the analysis of the Stringent Dataset in Study 1 (shown in Figure 3) were extracted for between 2 and 20 clusters. For example, a 2-cluster solution would create two clusters: Language-color and everything else. Thus, each person would be assigned a value of 1 or 2 indicating the number of types they have (a value of 0 is never found because everybody in the analysis has some recognized type). For a 3-cluster solution, the three types would be Language-Color, Language-Taste, and everything else. Participants would be assigned a value of 1, 2, or 3 depending on the number of clusters they have. Four clusters would consist of Language-color, Language-taste, Personification, and everything else (scores of 1, 2, 3, or 4), and so on. The scores for each person (number of clusters they have) are correlated with the questionnaire measures at each level of cluster extraction. The correlation between trait (CLaN, GSQ, Psi-Q) and a number of clusters of synesthesia a person has (at different levels of cluster extraction) is shown in Figure 7. Given the large volume of correlations performed, the resulting data can be interpreted as a descriptive set of effect sizes for which inferential (*p*-value) statistics are not needed but would, instead, guide future confirmatory research. The peak for our Psi-Q measure of mental imagery was at 5 clusters with a plateau between 5 and 7 clusters (a plateau was defined as a 5% difference in *r* around the peak). The peak for our GSQ measure of sensory sensitivity was at 12 clusters with a plateau between 7 and 17 clusters. For the CLaN phenomenology measure, there was an initial peak around 6 clusters (plateau at 6–16 clusters) but a global peak at 25 clusters (plateau of 23–43). As such, extracting ∼ 7 clusters would provide a satisfactory and more conservative description of the “dose effects” of synesthesia across three independent measures. Note that going up to 20 clusters does not fractionate the earlier extracted clusters but instead whittles down the unclustered/miscellaneous category.

**Figure 8.**
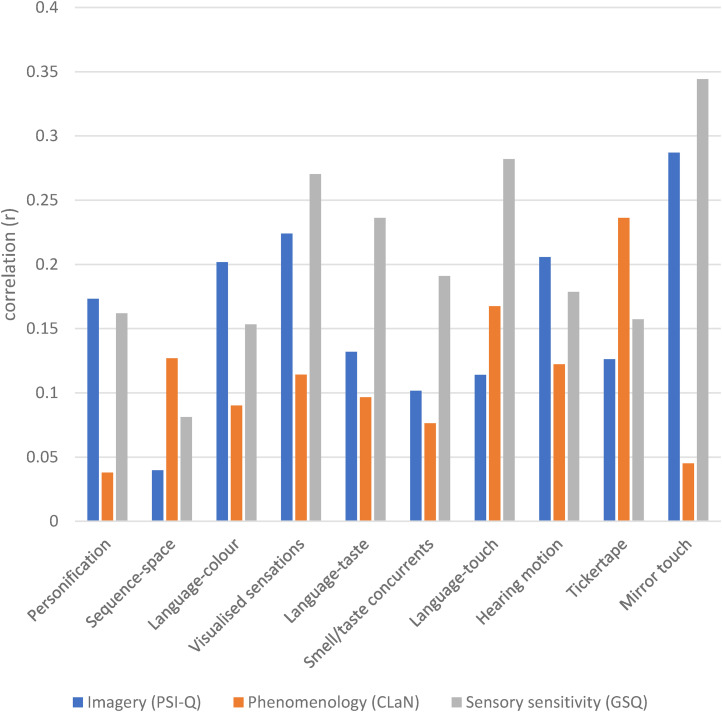
The size of correlation (*r*), *y*-axis, between questionnaire scores and the presence or absence (1 or 0) of particular clusters/types of synesthesia.

We point out that the above analysis is concerned solely with the number of clusters of synesthesia (e.g., whether a person has 1 out of five clusters or four out of five clusters) and not with the actual clusters themselves (e.g., whether a person has Visualized sensations or Language-taste). Here we explore whether different clusters behave in the same way by performing point biserial correlations in which the presence/absence of each cluster is considered separately. Following Study 1, we considered the first seven clusters together with the three most common remaining types (hearing-motion, tickertape, mirror-touch). The results are shown in Figure 8. Again, given the large number of correlations, these provide an overall picture of effect sizes rather than enabling an interpretation of individual correlations (which are numerous). The basic pattern is of small (*r* < .3) positive correlations across the three dependent variables irrespective of the cluster being considered. That is, it is unlikely that the previous analyses of dose effects were driven solely by the presence of one or two specific clusters having disproportionate impacts on the three questionnaire measures.

## General Discussion

In this study, we aimed to better understand the varied and complex phenomenology that makes up the experience known as synesthesia. It has been relatively unclear exactly how to splice up the “phenomenological space” of synesthesia or to understand how different types cluster together. Is the experience of colors from music in any way linked to colors from letters? Or do colors from letters better link with tastes from letters? How can we tell? To answer these questions we examined which types of synesthetic experiences group together within a single individual, and similarly, how these clusters might predict other traits found in synesthetes (e.g., heightened mental imagery or sensory sensitivity). We answered these questions using large cohorts of synesthetes, a number of which were given additional measures in imagery, sensory sensitivity, and synesthesia phenomenology. Firstly, we summarize the main clusters of synesthesia that we observed in Experiment 1. Here we compared them against the most relevant previous study of a similar type by Novich et al. (2011). Secondly, we discuss how many clusters of synesthesia there may actually be. Finally, we discuss the issue of independence or inter-dependence of clusters.

### How Do Types of Synesthesia Cluster Together?

In Study 1, we began with a maximum of 164 different associations between possible inducers (e.g., letters) and concurrents (e.g., colors). We defined these as “types” and then extracted “clusters” based on the strength of the correlations among them. The first cluster was termed “Language-Colour”; this closely resembles the cluster identified by Novich et al. (2011) which they termed “Coloured Sequences.” Our cluster also contains colored words and names, not included as potential inducers by Novich et al. (2011), hence our different choice of terminology. Both Novich et al. (2011) and our study started with a single category of Sequence-Space (made up of types such as days-space, number-space which were not separately entered into the analysis) and, in both studies, there was no further higher-order clustering (e.g., with other visual concurrents).

Our cluster termed “Visualised Sensations” closely resembles the one of the same name reported by Novich et al. (2011) which contains pairings with color concurrents such as pain-color, taste-color, smell-color, emotion-color. However, we also find that the concurrent experience tends to involve shapes as well as colors (e.g., pain-shape, smell-shape) which were not included as potential types by Novich et al. (2011). These synesthetic experiences are likely to have multiple visual elements—perhaps also extending to motion and texture (which neither study asked about). However, the most significant discrepancy is that we find that “Coloured hearing” (music-color, noises-color, voice-color) falls within this same cluster whereas Novich et al. (2011) reported it as a separate cluster (comprising chords-color, instrument-color, and music pitch—color). In our analysis, we would have to create as many as 40 clusters in the Stringent dataset before the Colored hearing would emerge as a separate entity. We do not know the origin of the difference between studies and it would be important for others to replicate with different stimulus material and participants. It is unlikely to be differences in statistical power because the metric used to form the clusters (*r*-values) is not dependent on sample size.

There is evidence that synesthesia involving taste/smell concurrents subdivide into two kinds depending on whether the inducers are linguistic or sensory (in our clusters named Language-Taste and Smell/Taste Concurrents, respectively). This resembles the same division observed for color (i.e., Language-Color and Visualized Sensations). It is also noteworthy that for the Language-color category the most common inducers are graphemes (letters and numbers), whereas for the Language-taste category the most common inducers are whole words (see, Simner et al., 2006; Simner et al, 2009; Simner & Haywood, 2009, for evidence that tastes emerge via lexical networks, while colors emerge via graphemic ones). There are also a small number of taste/smell concurrents in the cluster termed Non-visual Sequalae by Novich et al. (2011) but for their study, these occurred amongst a more heterogeneous set (vision–smell, vision–taste, sound–taste, sound–smell, sound–touch, vision–sound).

The clusters of Personification and Language-Touch that emerged in our analysis could not have been observed by Novich et al. (2011) because they were not part of the set of 22 types considered in that earlier study. Personification is widely considered a form of synesthesia, although the fact that the concurrent experience is not percept-like makes it something of an outlier. However, non-perceptual elements are common elsewhere in synesthesia notably in the stimuli that act as inducers (Jürgens & Nikolić, 2012).

One important characteristic of our seven clusters that emerges from our analysis is that clusters are more strongly unified by a common concurrent than by common inducers. This is a non-trivial fact because, from the first principles, one could imagine a cluster such as number-color/number-taste/number-touch (i.e., united by numerical inducers) but this was not generally observed. Instead, clusters revolve around shared concurrents (e.g., pain-color and smell-color in the same cluster). This asymmetry between inducer and concurrent raises important questions about the neurological roots of synesthesia. Where unusual connections or disinhibited pathways are assumed to contribute to synesthesia (Bargary & Mitchell, 2008) these appear to be feeding back from concurrent regions, rather than initiating from inducer areas. For Visualized sensations, for example, the visual system may act as a kind of “homing beacon” during development such that it is able to initiate and stabilize long-range connectivity with other sensory centers. Although some role of learning in synesthesia is inevitable (e.g., linguistic inducers are learned), it is harder to imagine how the clusters observed in this research emerge solely from learning based on environmental contingencies.

Mirror-touch, hearing-motion, and tickertape synesthesia all have the characteristic of appearing to be lone “islands” that do not correlate strongly with and hence do not cluster with, other types of synesthesia. (Sequence-space has moderate correlations with “days-shape” and “months-shape” but these latter two pairings are likely referring to the former in any case). However, it may be that our method was not suited to revealing appropriate clusters. Let us take, for example, personifications (e.g., A is female) and mirror-touch synesthesia (e.g., feeling touch on the body when watching another person being touched). Importantly, in the case of Personification, we presented multiple examples of inducers (letters, numbers, days, months) but in the case of mirror-touch, we presented a single example only (not hands, faces, legs separately). This is undeniably true but, in all cases, one can still imagine plausible clusters that could have emerged. Mirror-touch could have clustered with other tactile concurrents (e.g., music-touch). Tickertape (seeing visualized words from speech) could have clustered with the Language-color or Visualized sensations. Hearing-motion could have clustered with other auditory concurrents. The fact that this did not happen leaves them with an uncertain status of being either standalone types of synesthesia, or not types of synesthesia at all. However, other evidence suggests that they belong within the same synesthesia umbrella. They show the same “snowball effect” (Study 1) and show external validity in predicting traits linked to synesthesia (Study 2).

### How Many Clusters are There?

It is hard to give a definitive answer to this question from the evidence available. This question comes down to how much each new cluster accounts for variance in the data. If adding another cluster makes little difference, then should we consider that a cluster at all? This is often a judgment call. Nonetheless, we agree with Novich et al. (2011) that the answer is likely to be closer to their estimate (*N*  =  5) than the large number of observed inducer-concurrent pairings that have been reported as individual types (e.g., *N*  =  60 in Day, 2005). Here we offer the tentative solution of up to 10. These consist of seven true clusters (i.e., each containing multiple types) and three common “islands” each comprising a single type (mirror-touch, tickertape, hearing-motion). (Sequence-space could also be considered an island rather than a cluster, but this is essentially a point of terminology and how we opted to measure it).

One could make a case for considering around five subtypes by dropping some of the rarer and less well-documented varieties (e.g., Language-touch) and some of the varieties which people have doubted are causally linked to synesthesia such as mirror-touch (Rothen & Meier, 2013). But there is a risk in relying on intuition rather than evidence. If the aim is to group synesthetes as having few or many clusters then adopting this more conservative approach is likely to make minimal difference in practice because of the snowball effect that we observed. If we ask about five clusters of synesthesia and find someone with four (out of a possible five) and someone with one then the difference between these synesthetes is unlikely to disappear by considering further undisclosed clusters. Instead, the difference is likely to grow even wider. Conversely, one could make a case for going beyond ten clusters but it’s not clear what the candidate subtypes would be. Almost certainly, they are going to be rare and, hence, not have much influence.

### How Independent are the Clusters? What Causes the Clusters to Emerge?

Above we considered how types of synesthesia merge into clusters. Let us now consider the ways in which different clusters correlate with each other. The process of creating clusters (or factors) necessarily tries to maximize the similarity within the cluster and minimize the similarity between clusters. Nevertheless, a residual association is found in terms of weak positive correlations amongst all clusters (*r* of about + 0.1). Based on a similar pattern of results, Novich et al. (2011) made the claim that different clusters of synesthesia may have different causes (e.g., different genes). However, the claim made here is that these weak statistical associations could mask the true degree of association amongst subtypes.

The analyses conducted here (and similarly for Novich et al., 2011) do not take into account the prevalence of synesthesia in the general population. This is crucial for determining the population-level degree of association between clusters. We can simulate the effect of adding non-synesthetes to our correlation analyses: non-synesthetic controls have a value of zero for every possible cluster of synesthesia. For every 1 synesthete, we can simulate the effect of adding 5 or 10 or 20 controls which are reasonable estimates (e.g., Fassnidge et al., 2017, reports a prevalence of one in five for hearing-motion; Ward et al., 2018b, reports a prevalence of 1 in 10 for sequence-space; and Simner et al., 2006, report a prevalence of 1 in 20 predominantly for language-color). Here, in the combined samples, the average correlation between clusters jumps from *r*  =  .092 (0 controls) to *r*  =  .644 (5: 1 ratio of control: synesthete) to *r*  =  .663 (10: 1 ratio of control: synesthete) to *r*  =  .672 (20: 1 ratio of control: synesthete). That is when we model synesthesia at a population level (including both synesthetes and controls) we see that the size of the association goes up considerably.

A seemingly weak correlation between two clusters of synesthesia may also mask the degree of association if there is a snowball effect: that is, the degree of association between clusters increases according to the overall number of clusters possessed. To consider an analogy: imagine that we toss nine coins. If we get only one head out of nine, then what is the probability we’d get another head on a tenth coin toss? The answer should be *p*  =  .5. In contrast, if we get six heads after tossing nine coins, then what is the probability that we’d get another head on a tenth toss? The answer should still be *p*  =  .5. Synesthesia does not work like this. If a person has only one known cluster of synesthesia (out of nine counted) then they are far less likely to have some additional tenth cluster than a person with six known clusters (out of nine)—the probabilities are .2 and .6, respectively, so a threefold increase in probability. In effect, the number of times synesthesia is observed in an individual constitutes evidence for a greater degree of bias in the system. In the coin analogy, it would be as if the number of heads we observe constituted increasing evidence for head-weighted coins rather than reflecting some chance combination of events. In the case of synesthesia, we assume that this weighting/bias reflects the increasing influence of some latent variable that causes synesthesia to emerge in some people and not others (Ward, 2019b). Moreover, it does not matter which nine types we count and which the tenth type we leave out because a snowball effect is found in all cases. This in itself is suggestive evidence that all of these 10 clusters could be considered as being related (i.e., having a common cause).

### Limitations: Truthful Responding, Acquiescence Bias, and Sampling Bias

One immediate limitation is that there is no independent measure of the truthfulness of these self-reported types of synesthesia in Study 1. In particular, there is a bias in psychological research for people to choose “agree” or “yes” options—termed an acquiescence bias. Although we primarily use checkboxes rather than statements, the problem may still arise. To minimize this, it is to be noted that the Stringent dataset was trimmed to remove people who reported many implausible types of synesthesia that would be indicative of an acquiescence bias. On average, our synesthetes are far more likely to *deny* having types of synesthesia than admit to them. The mean number of clusters endorsed (out of 10) is 3.5, with only 15.2% reporting types that fall into six or more clusters and only 2.9% reporting having eight or more. That is, whilst we can’t rule some influence of acquiescence bias we attempted to minimize this, and the sample as a whole is quite selective in the statements that they endorse. Furthermore, the clusters we established in a self-referred sample in Study 1 showed validation from the traits we measures in Study 2 (imagery, sensitivity, phenomenology).

Practical constraints mean it is not possible to ascertain the veracity of all the kinds of synesthesia reported by every synesthete we tested in Study 1. However, some recent research points to some stability in the pattern of responding insofar as we have been able to look for it. Ward (2019a) noted that the MTS screening question on the questionnaire predicted performance on a longer test of MTS administered, on average, three years later. The hearing-motion question on the screening questionnaire did not predict subsequent performance on the MTS test.

Finally, there is a concern that the synesthetes we have observed in this study (who have contacted our research group via the internet) may not be representative of the synesthesia community as a whole. This could manifest itself in several ways. One possibility is that the types themselves might present as a different pattern. For example, maybe number-color and number-taste would emerge as a cluster from a truly representative sample (even though it did not here). However, this seems unlikely because if such people did exist there is no reason why they would be less likely to come forward. A more realistic concern is that the mean number of clusters that synesthetes possess is lower than the value of 3.5 that we report: that is, there is a bias for people with more kinds of synesthesia to volunteer for research (see, Simner, 2012, for a similar argument). This seems reasonable and it would be important for future research to revisit this. The most reliable prevalence study to date did not include some of the most common types of synesthesia including mirror-touch, sequence-space, personification, tickertape, and hearing-motion (Simner et al., 2006). Hence, we do not have a clear picture as to how frequently these clusters gravitate together in the general population, and our only window into this has come via the self-referral of synesthetes.

In summary, we have shown that inducers and concurrents do not pair randomly within synesthesia. Instead, we found that having one type of pairing (e.g., music-color) raises the likelihood of further specific types (e.g., emotion-color) over others (e.g., word-taste). This means that synesthesias cluster together within individuals, and the number of clusters within any given person also predicts other traits, such as the vividness of their mental imagery or the degree of their sensory sensitivity. It is hard to give a definitive answer as to how many clusters of synesthesia exist, although our data offers an evidence-based judgment call. We place this number at approximately 7, but perhaps no more than 10.

## Supplemental Material

sj-docx-1-pec-10.1177_03010066211070761 - Supplemental material for How do Different Types of Synesthesia Cluster Together? Implications for Causal Mechanisms Click here for additional data file. Supplemental material, sj-docx-1-pec-10.1177_03010066211070761 for How do Different Types of Synesthesia Cluster Together? Implications for Causal Mechanisms by Jamie Ward and Julia Simner in Perception

sj-xlsx-2-pec-10.1177_03010066211070761 - Supplemental material for How do Different Types of Synesthesia Cluster Together? Implications for Causal Mechanisms Click here for additional data file. Supplemental material, sj-xlsx-2-pec-10.1177_03010066211070761 for How do Different Types of Synesthesia Cluster Together? Implications for Causal Mechanisms by Jamie Ward and Julia Simner in Perception
