# Supplementary material for: How do Different Types of Synesthesia Cluster Together? Implications for Causal Mechanisms
Source: Perception. 2022 Jan 18;51(2):91–113. doi: 10.1177/03010066211070761 (PMC8811335; doi:10.1177/03010066211070761)
Supplement: sj-docx-1-pec-10.1177_03010066211070761 - Supplemental material for How do Different Types of Synesthesia Cluster Together? Implications for Causal Mechanisms [file sj-docx-1-pec-10.1177_03010066211070761.docx]

**Supplementary Material**

Supplementary Methods

*Figure S1. A screenshot of the grids in which participants reported their types of synaesthesia*
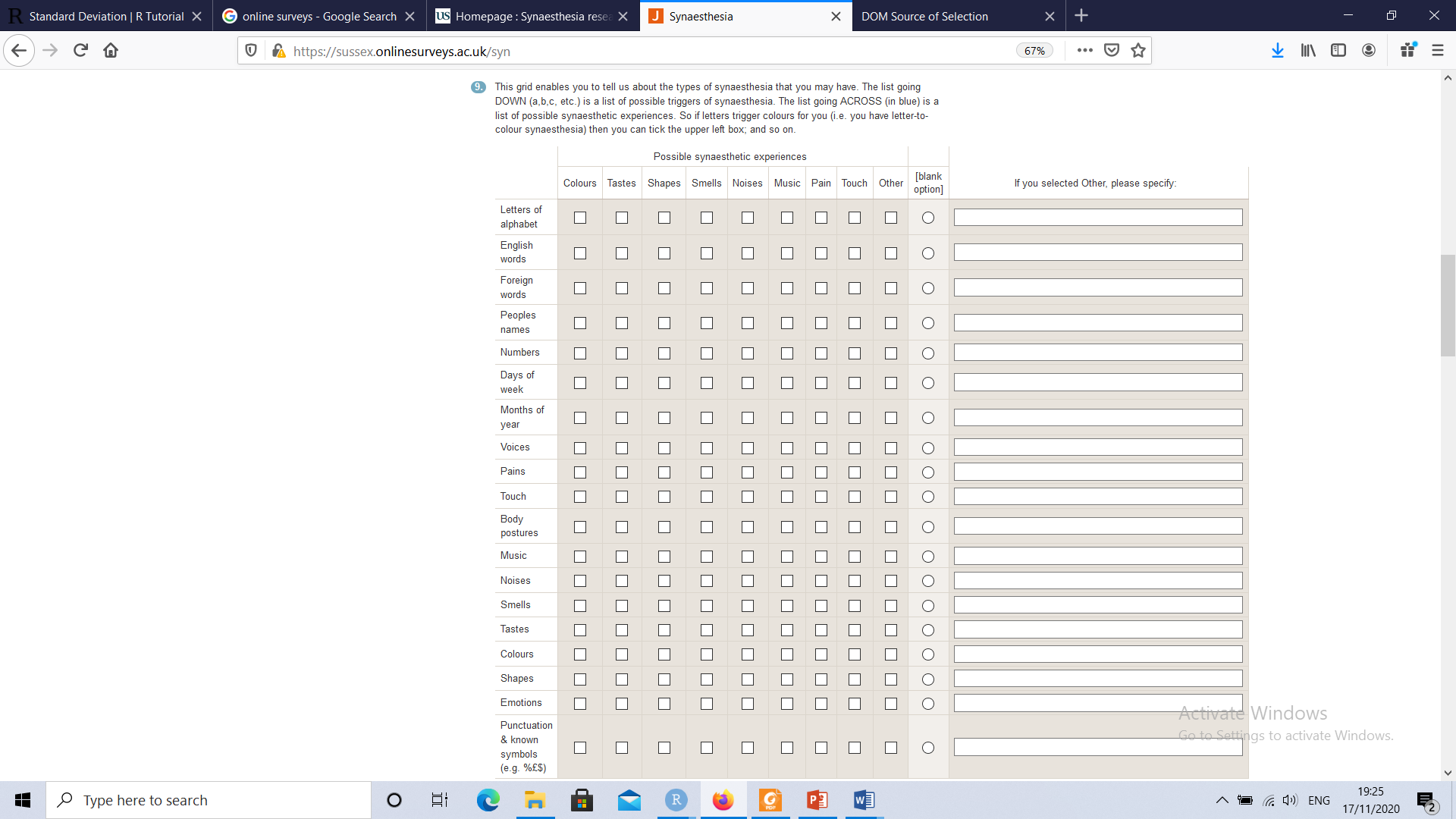

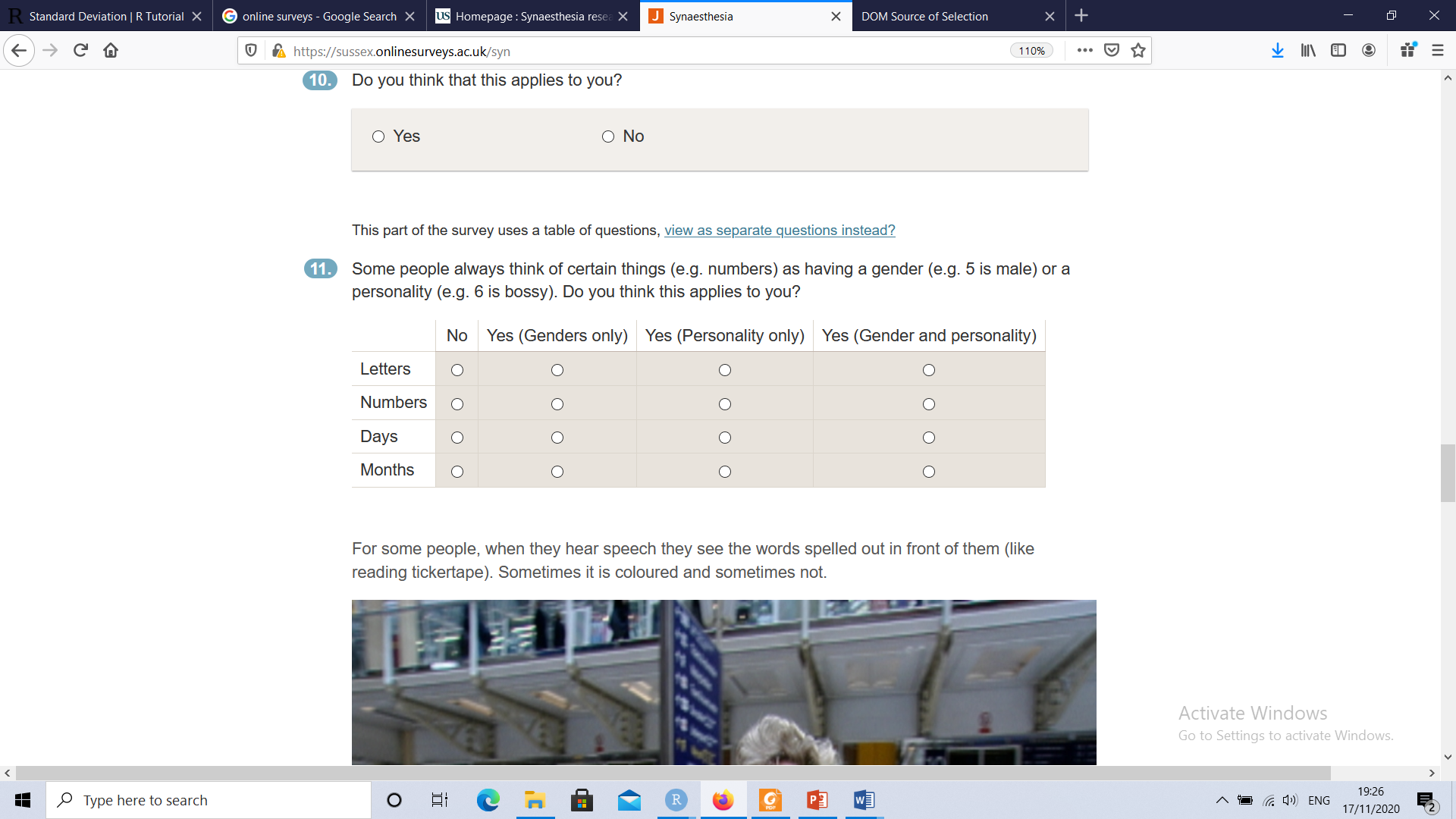


*Figure S2. Images used in the screening questionnaire to illustrate sequence-space synaesthesia (top and middle) and tickertape synaesthesia (bottom). The links to the videos for mirror-touch and hearing-motion are* [*https://youtu.be/aoUdvuLrawE*](https://youtu.be/aoUdvuLrawE) *and* [*https://youtu.be/o39TiACe4mw*](https://youtu.be/o39TiACe4mw)


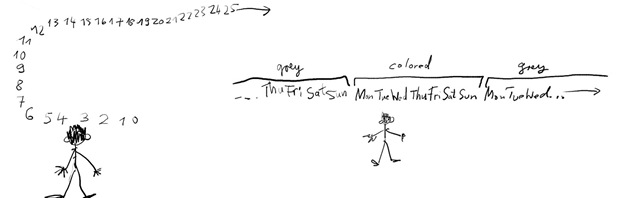


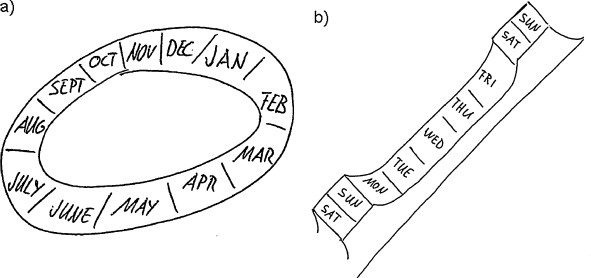


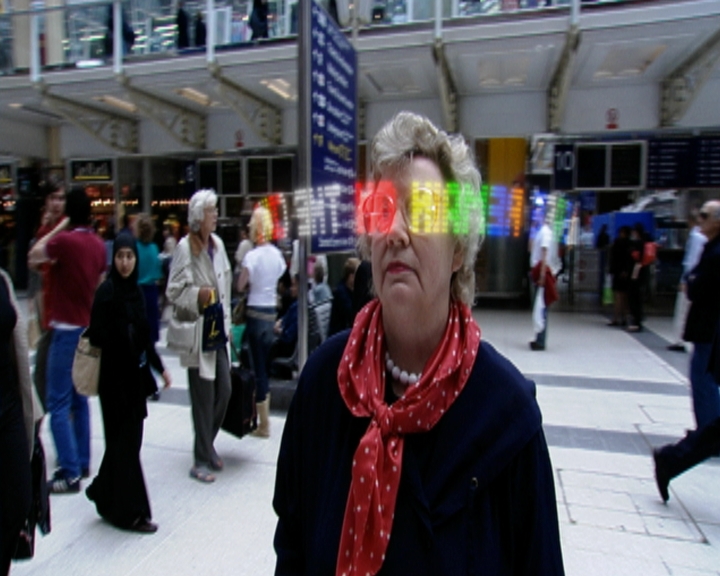


Supplementary Results

*Figure S3. Full dendrogram of all N=164 types of synaesthesia in the Inclusive dataset. The colours and labels for the different branches reflect the judgment of the authors.*


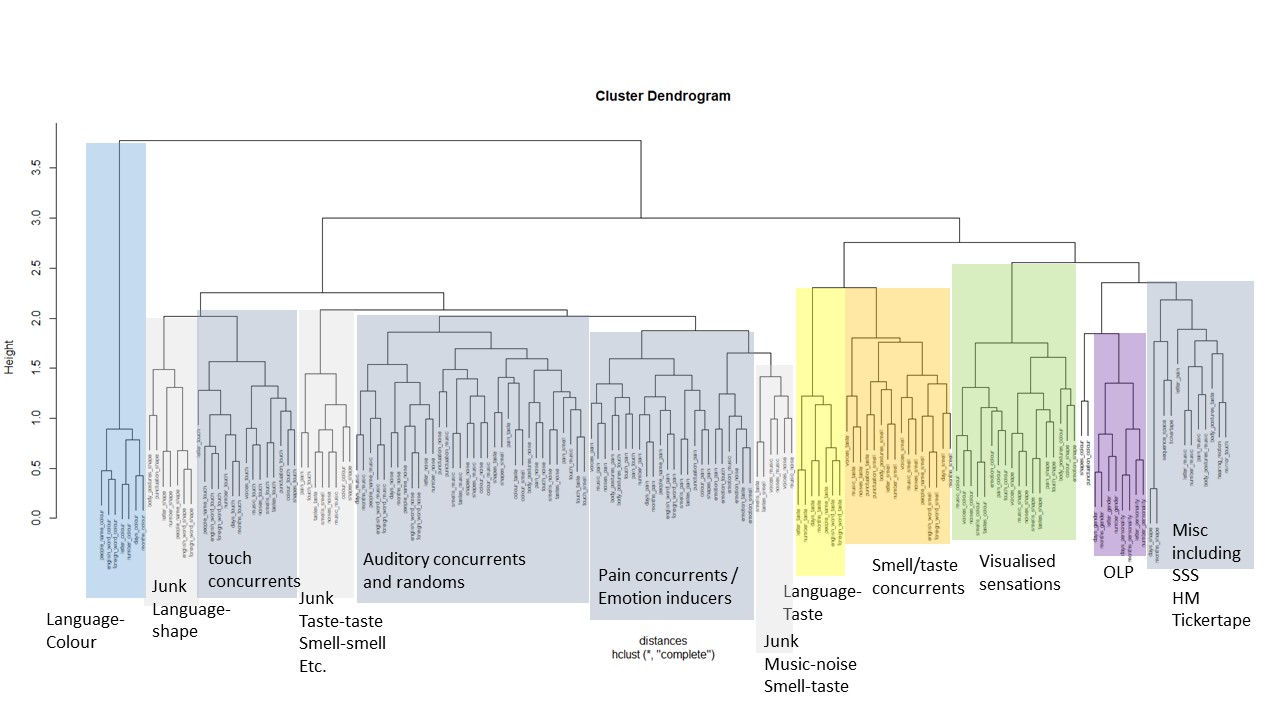


*Figure S4. Full dendrogram of all N=112 types of synaesthesia in the Stringent dataset. The colours and labels for the different branches reflect the judgment of the authors. The ‘cut lines’ show how different numbers of clusters are obtained.*


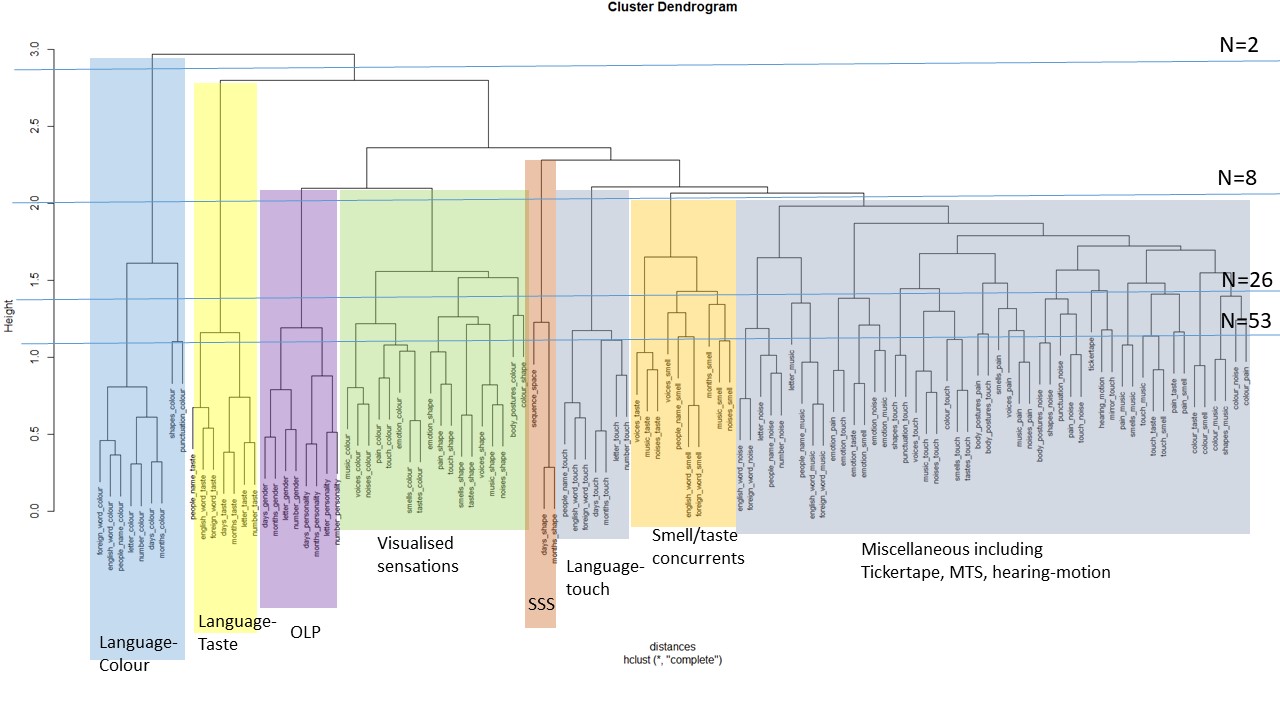


Table S1.

This is in an accompanying excel file called ‘factor analysis 21 factors.xlsx’.

The first sheet contains the full result of the factor analysis (21 factors shown in columns and 112 rows for each type of synaesthesia). The numbers represent factor loadings.

The second sheet contains a simplified version of the factor analysis (21 factors shown in columns and 95 rows for each type of synaesthesia with a factor loading > .3). The numbers represent factor loadings and only factor loadings >.3 are displayed.
